# Supplementary figures and images for: A high-content screen identifies the vulnerability of MYC-overexpressing cells to dimethylfasudil
Source: PLoS One. 2021 Mar 24;16(3):e0248355. doi: 10.1371/journal.pone.0248355 (PMC7990233; doi:10.1371/journal.pone.0248355)

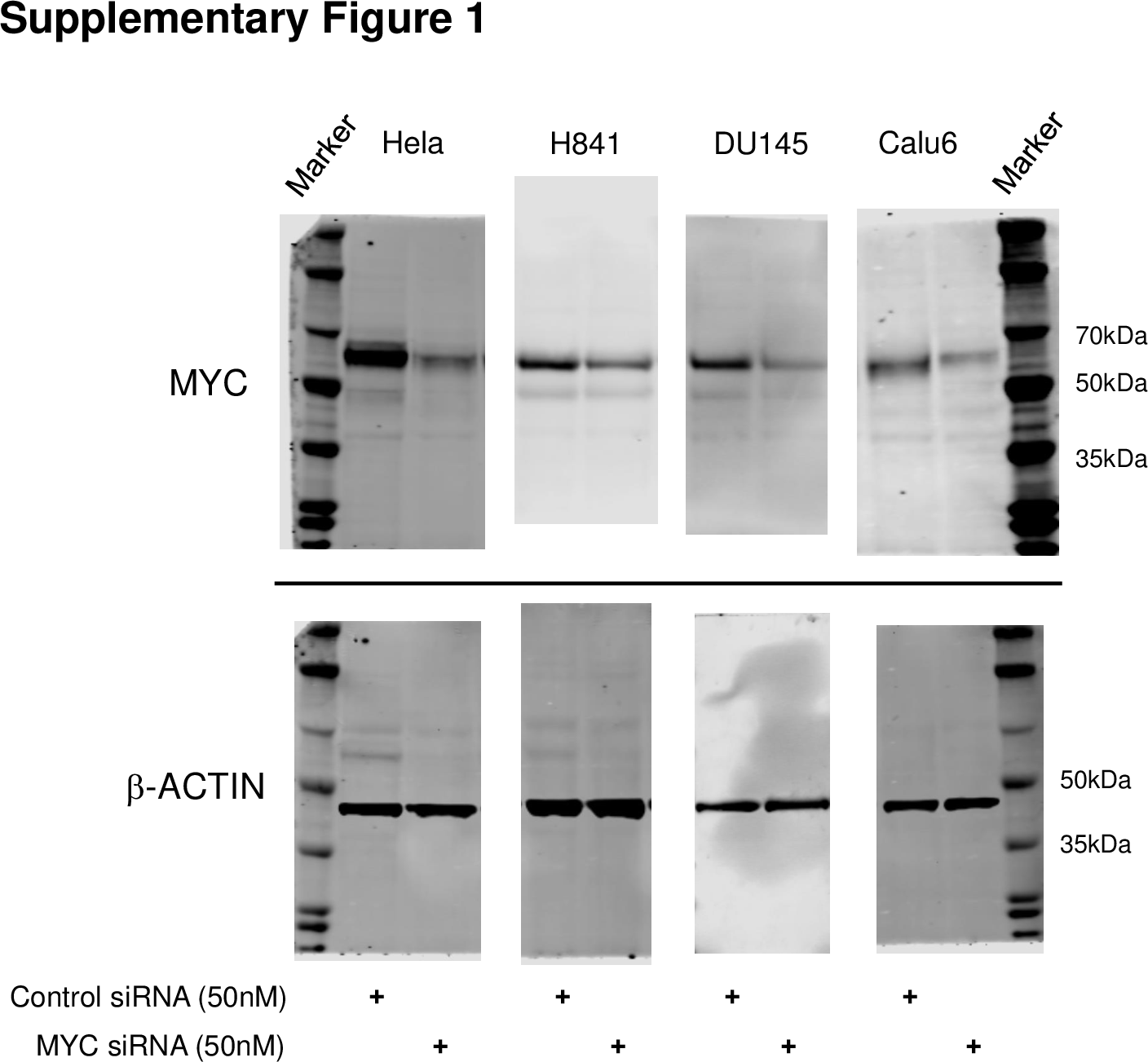

Supplement: S1 Fig — The cell lines indicated were treated with control siRNA oligonucleotide or MYC siRNA oligonucleotide, both at a concentration of 50 nM. Levels of MYC (top) and β-ACTIN (loading control, bottom) were assayed by Western analysis to demonstrate knockdown. Band capture is presented in Fig 4B. (TIF) [file pone.0248355.s001.tif]
